# *Fusobacterium necrophorum* Pharyngitis Complicated by Lemierre's Syndrome

**DOI:** 10.1155/2016/3608346

**Published:** 2016-12-06

**Authors:** Antonio Faraone, Alberto Fortini, Gabriele Nenci, Costanza Boccadori, Valerio Mangani, Roberto Oggioni

**Affiliations:** ^1^Internal Medicine, “San Giovanni di Dio” Hospital, Via di Torregalli 3, Florence, Italy; ^2^Intensive Care Unit, “San Giovanni di Dio” Hospital, Via di Torregalli 3, Florence, Italy

## Abstract

We report the case of an 18-year-old woman who was referred to our outpatient clinic because of a 2-week history of sore throat, high fever, and neck tenderness unresponsive to a 7-day amoxicillin/clavulanic acid course. Infectious mononucleosis was initially suspected, but an extremely high value of procalcitonin and clinical deterioration suggested a bacterial sepsis, prompting the patient admission to our internal medicine ward. Blood cultures were positive for* Fusobacterium necrophorum*. CT scan detected a parapharyngeal abscess, a right internal jugular vein thrombosis, and multiple bilateral lung abscesses, suggesting the diagnosis of Lemierre's syndrome. The patient was treated with a 2-week course of metronidazole and meropenem with a gradual clinical recovery. She was thereafter discharged home with metronidazole and amoxicillin/clavulanic acid for 14 days and a 3-month course of enoxaparin, experiencing an uneventful recovery. The present case highlights the importance of taking into consideration the Lemierre's syndrome whenever a pharyngotonsillitis has a severe and unusual course.

## 1. Introduction

Acute febrile sore throat is usually due to a viral or bacterial pharyngotonsillitis that resolves in a few days without clinical complications. Occasionally, the clinical course is much more severe: we describe the case of a young woman with a life-threatening sepsis complicating a pharyngeal infection by* Fusobacterium necrophorum*.

## 2. Case Report

**Figure 1:**
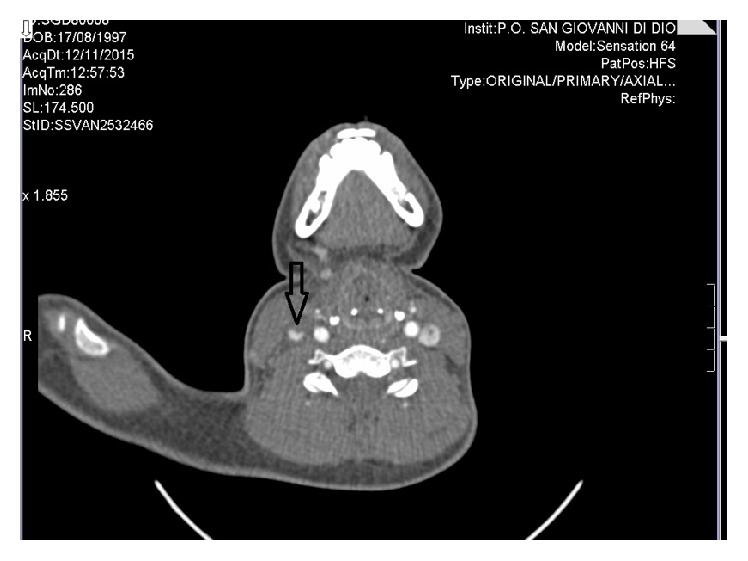
CT scan of the neck which shows (arrow) a filling defect of the right internal jugular vein due to partial thrombotic occlusion.

**Figure 2:**
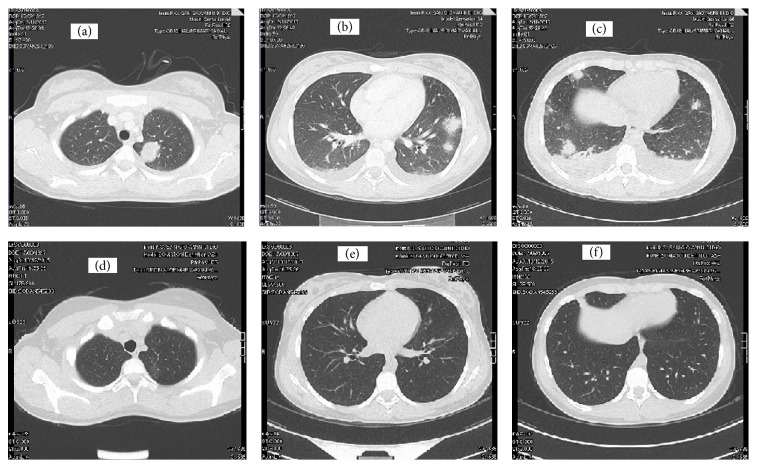
(a, b, c) CT scan of the thorax demonstrating bilateral nodular lesions and bilateral pleural effusion. (d, e, f) CT scan after 4-week antibiotic therapy which shows the full resolution of the nodular lesions and pleural effusion.

An otherwise healthy 18-year-old woman was referred to our outpatient clinic because of a 2-week history of sore throat, high fever, and mild neck tenderness. Ten days before, she had been diagnosed with acute pharyngotonsillitis and had started a 7-day amoxicillin/clavulanic acid course (1 g bid), obtaining only partial relief of the throat pain. On examination, the patient was febrile (38.2°C); neck palpation revealed tender lymph nodes in the right anterior cervical chain. The tonsils were enlarged but not erythematous; breath sounds were diminished over the right lung base. Examination of the abdomen revealed slight splenomegaly. The diagnostic suspicion was oriented toward infectious mononucleosis and, after the collection of blood samples for laboratory tests, the patient was discharged home with the prescription of paracetamol. Laboratory tests showed neutrophilic leukocytosis (white blood cells count 11.56 × 10^9^/L, neutrophils 93%), low platelet count (61.00 × 10^9^/L), and elevated C-reactive protein (25.9 mg/dL). Serological tests for EBV, CMV, and HIV were negative. An unexpectedly high procalcitonin value (294 ng/mL) raised the suspicion of bacterial sepsis and elicited an urgent hospitalization in our internal medicine ward for diagnostic and therapeutic management. On presentation, the patient was alert, hypotensive (BP 100/60 mmHg), and tachycardic (115 bpm); body temperature was 38.7°C; respiratory rate was 17, with oxygen saturation of 97% on room air. A chest X-ray showed a faint retrocardiac opacity and raised the suspicion of a community acquired pneumonia. After drawing two sets of blood culture (aerobic, anaerobic), an empirical treatment with intravenous ceftriaxone and azithromycin was started. Two days later, the patient was still febrile and mildly lethargic, with toxic appearance. Blood cultures grew gram negative bacilli, and amikacin was added to ceftriaxone. The next day,* Fusobacterium necrophorum*, an anaerobic gram negative rod, was identified as responsible for the bloodstream infection, and the antibiotic treatment was changed to intravenous metronidazole and meropenem. Based on this microbiological finding, a neck ultrasound exam was performed, showing a nonoccluding parietal thrombosis of the right internal jugular vein. Diagnosis of Lemierre's syndrome was ultimately confirmed by a contrast enhanced CT scan of the neck and the thorax. This showed a small right parapharyngeal abscess, thrombosis of the right internal jugular vein (Figure 1), and multiple peripheral lung nodules, expression of septic embolization (Figures 2(a), 2(b), and 2(c)). Anticoagulation with subcutaneous enoxaparin was prescribed (4,000 UI bid); subsequently, the patient was transferred to the High Dependency Unit for haemodynamic monitoring. Five days later, the body temperature became steadily normal and blood tests showed a sharp decline of procalcitonin (2.11 ng/mL) and C-reactive protein (2.3 mg/dL), an increase of platelet count (428.00 × 10^9^/L), and the negativization of microbial cultures. The patient was discharged home after a 2-week course of intravenous antibiotic treatment. She received an outpatient oral treatment with metronidazole and amoxicillin/clavulanic acid for 14 days and a 3-month course of enoxaparin, experiencing an uneventful recovery. A control CT scan showed the complete resolution of lung opacities and pleural effusion (Figures 2(d), 2(e), and 2(f)).

## 3. Discussion

Lemierre's syndrome is a rare but life-threatening disease that mainly affects healthy young patients [1, 2]. It appears as an oropharyngeal infection complicated by sepsis, jugular vein thrombosis, and septic embolization to lung and other organs.* Fusobacterium* species, most commonly* F. necrophorum*, are responsible for the majority of cases [3]. Up to one-third of patients presents a mixed infection, with frequently present streptococci and other gram negative anaerobes.* F. necrophorum* is a non-spore-forming gram negative anaerobic rod belonging to the normal flora of the oropharynx and is recognized as the causal agent of approximately 10% to 20% of pharyngitis cases in adolescents [3–6]. This condition was more frequently encountered in preantibiotic era; it subsequently became a rare disease, but recent reports have documented an increasing number of cases worldwide [7–9]: a possible explanation for this recrudescence is the reduction in the empirical use of antibiotics in patients with sore throat, following the advice of clinical guidelines [10]. However, there is no clear evidence that Lemierre's syndrome is more frequent when antibiotics are prescribed to a lesser extent [11]. In the present case, an empirical course of antibiotics, even active against* F. necrophorum* (amoxicillin/clavulanic acid), was early prescribed, but the dose and treatment duration were probably insufficient.

The diagnosis of Lemierre's syndrome requires full awareness of this rare condition and high index of clinical suspicion. The disease should be suspected in young patients with history of oropharyngeal infection who failed to improve either spontaneously or after antibiotic treatment and developed clinical and laboratory evidences of sepsis, respiratory symptoms, or atypical unilateral neck pain and swelling. The latter symptoms are characteristic of Lemierre's syndrome, being the consequence of unilateral jugular vein thrombosis. Confirmation of the diagnosis requires the detection of the internal jugular thrombophlebitis and septic embolization to lung and other organs by imaging studies (duplex ultrasound, computed tomography, and magnetic resonance) and the isolation of* Fusobacterium* species from blood cultures.

The recommended antibiotic therapy includes metronidazole plus another molecule active against streptococci and other coinfecting pathogens of the oral cavity [1]. Metronidazole is bactericidal and has an excellent penetration into most tissues, including tonsils. Most commonly, metronidazole is associated with a beta-lactam antibiotic such as amoxicillin/clavulanic acid or ceftriaxone, but the use of penicillin, clindamycin (particularly in case of penicillin allergy), and meropenem is also frequently described [1]. The recommended duration of antibiotic therapy is 4–6 weeks [1, 3]. Considering the severity of the clinical picture, the possible polymicrobic infection and the initial lack of efficacy of amoxicillin/clavulanic acid, we decided to use metronidazole in combination with a broad spectrum antibiotic such as meropenem.

A critical reappraisal of the case suggests that an empirical antibiotic therapy with metronidazole should have been started even before obtaining the microbiological response from blood cultures, bearing in mind the possibility of an anaerobic infection of the pharynx. Similarly, a neck imaging study to search a jugular thrombophlebitis and/or neck abscesses had to be performed earlier.

Anticoagulation remains controversial, but it is recommended when there is evidence of thrombus propagation, septic embolization, or unsatisfactory clinical response to antibiotics alone [3, 12]. We empirically decided to use enoxaparin (4000 UI sc bid) without evidence of thrombus propagation and side effects.

## 4. Conclusions

We reported the case of a young woman with Lemierre's syndrome due to* Fusobacterium necrophorum* infection. The appropriate therapy was introduced only after the isolation of the microorganism from blood cultures. The present case suggests that (1) the recommended oral antibiotic treatment for acute pharyngeal infections does not always prevent the progression of the disease into the life-threatening Lemierre's syndrome; (2) an anaerobic infection and the Lemierre's syndrome should be suspected in patients affected by a pharyngotonsillitis with a severe and unusual course; (3) in these patients an empirical antibiotic therapy, active also against anaerobic microorganism, has to be started early; moreover, an imaging study of the neck, searching for jugular thrombophlebitis and parapharyngeal abscesses, should be obtained as soon as possible.
